# Sex differences in nicotine-enhanced Pavlovian conditioned approach in rats

**DOI:** 10.1186/s13293-019-0244-8

**Published:** 2019-07-17

**Authors:** Sierra J. Stringfield, Aric C. Madayag, Charlotte A. Boettiger, Donita L. Robinson

**Affiliations:** 1 Bowles Center for Alcohol Studies, University of North Carolina, CB #7178, Chapel Hill, NC 27599-7178 USA; 2 Neuroscience Curriculum, University of North Carolina, Chapel Hill, NC USA; 3 Department of Psychology and Neuroscience, University of North Carolina, Chapel Hill, NC USA; 4 Department of Psychiatry, University of North Carolina, Chapel Hill, NC USA

**Keywords:** Nicotine, Sex differences, Pavlovian conditioning, Sign tracking, Goal tracking, BDNF

## Abstract

**Background:**

Nicotine exposure enhances Pavlovian conditioned approach (PCA), or the learned approach to reward-predictive cues. While females show elevated approach to conditioned stimuli compared to males, potentially indicating heightened addiction vulnerability, it is unknown how sex may interact with nicotine to influence approach behavior. Additionally, brain-derived neurotrophic factor (BDNF) levels can be altered significantly after repeated nicotine exposure, suggesting a potential mechanism contributing to nicotine-induced behavioral phenotypes. The present study investigated the role of sex on nicotine-induced changes to stimulus-response behavior and associated BDNF protein levels.

**Methods:**

Male and female rats were exposed to nicotine (0.4 mg/kg, subcutaneously) or saline 15 min prior to each PCA session. PCA training consisted of 29 sessions of 15 trials, in which a 30-s cue presentation ended concurrently with a sucrose reward (20% *w*/*v* in water, 100 μL), and a 120-s variable intertrial interval occurred between trials. Approach behavior to the cue and reward receptacle was recorded. Preference toward the reward receptacle indicated a goal-tracking phenotype, and preference toward the cue indicated a sign-tracking phenotype, demonstrating that the cue had gained incentive salience. Twenty-four hours after the last PCA session, brain tissue was collected and BDNF levels were measured in the basolateral amygdala, orbitofrontal cortex, and nucleus accumbens using Western blot analysis.

**Results:**

Nicotine exposure enhanced both sign- and goal-tracking conditioned approach, and females showed elevated sign-tracking compared to males. There were no sex-by-drug interactions on conditioned approach. Day-to-day variability in conditioned approach was similar between sexes. In contrast to prior studies, neither repeated exposure to nicotine nor sex significantly affected BDNF expression.

**Conclusions:**

Drug-naïve females exhibited heightened sign-tracking compared to males, and nicotine enhanced conditioned approach similarly in males and females. Further, non-significant changes to BDNF expression in brain regions highly associated with PCA indicate that BDNF is unlikely to drive nicotine-enhanced conditioned behavior.

## Background

Repeatedly pairing an environmental stimulus with a reward (unconditioned stimulus, US) can lead to the formation of a stimulus-reward association through Pavlovian mechanisms. This changes the previously neutral environmental stimulus to a conditioned stimulus (CS) that can induce conditioned responses (CRs), such as approach [1]. In rodent models of Pavlovian conditioning using a CS that is localized in the test chamber and can be accessed by the animal, two classes of approach behavior typically emerge: sign-tracking CRs, in which animals approach and interact with the CS; and goal-tracking CRs, in which they approach and interact with the location of eventual US delivery [2, 3].

Expression of sign-tracking CRs suggests that the CS has become an incentive stimulus, as it is able to attract attention and motivate approach [4, 5]. In animals that exhibit high levels of sign-tracking, the CS also acquires conditioned reinforcing properties, in that the animals will perform instrumental responses to obtain the conditioned cue [6]. Animals that are categorized as sign-trackers display enhanced drug self-administration and are more likely to show other behaviors associated with vulnerability to addiction [3, 7, 8]. Rats bred to exhibit a “high responding” phenotype, in that they show increased locomotor response in a novel environment, are also likely to sign-track [9]. Sign-trackers and goal-trackers vary in terms of neurotransmitter release and neuronal activation associated with a preferred CR, suggesting that they are both acquired and heritable biological factors underlying the expression of these CRs [10–14].

Addictive drugs have been shown to increase the expression of sign-tracking behavior toward both drug- and non-drug-associated cues [5, 15, 16]. Nicotine, in particular, exerts reinforcement- and incentive-enhancing properties in both humans and animals, suggesting that nicotine amplifies the rewarding or incentive properties of non-nicotine stimuli [17–20]. For example, nicotine increases CRs to a non-drug-associated CS [18, 21, 22], especially sign-tracking CRs [16, 23, 24].

While investigating the effect of nicotine on behavior, we should consider the potential for variation in response due to sex. Most studies of Pavlovian conditioning that investigate both sign-tracking and goal-tracking CRs have used only male animals, but when females were included, moderate differences in CRs have emerged. For example, females are faster to acquire sign-tracking, show more conditioned reinforcement of a lever CS [25, 26], and show increased goal-tracking [27]. While the influence of nicotine on sex differences in Pavlovian CRs has yet to be established, some studies using animal models of nicotine self-administration indicate that females acquire nicotine self-administration faster than males [28, 29], while others find no difference between sexes [30, 31]. In terms of the relationship between nicotine exposure and the reinforcing properties of nicotine-associated cues, female rats respond more than males for nicotine reinforcement in the presence of a nicotine-paired stimulus [32]. In humans, female smokers are more sensitive to nicotine-associated stimuli than to the pharmacological effects of nicotine, while the opposite may be true in males [33]. Given this potential divergence between sexes in the influence of nicotine-associated cues, it follows to consider whether sex interacts with nicotine to amplify the conditioned motivational properties of otherwise inert cues. Using Pavlovian conditioned approach, we can investigate the possibility that females are more sensitive than males to the ability of nicotine to enhance conditioned responding toward a non-drug-associated stimulus, and thereby develop a greater understanding of the neurobiological basis of sex-dependent nicotine effects on behavior.

One mechanism by which nicotine may influence the incentive salience of non-drug-associated cues is through brain-derived neurotrophic factor (BDNF), a protein that modulates synaptic plasticity and has been associated with psychiatric disorders, behavioral responses, and drug abuse [34–36]. BDNF has been linked to conditioned responses, as drug-naïve sign-tracking rats exhibit reduced BDNF in the prefrontal cortex, but not the amygdala or striatum, compared to drug-naïve goal-tracking rats [37]. In addition, knockdown of prefrontal cortical BDNF using genetic mouse models influences both conditioned place preference and habitual behavior [38, 39]. BDNF has also been linked to nicotine: in humans, single-nucleotide polymorphisms associated with variation in BDNF expression have been associated with nicotine use, craving, and withdrawal [40–43]. Moreover, rodent studies demonstrate that both chronic and acute nicotine exposure can modulate BDNF levels [36, 44]. For example, BDNF levels in the striatum and hippocampus can either be increased [45, 46] or reduced [47, 48], depending on the duration of nicotine exposure. Thus, BDNF expression in key corticolimbic brain regions appears to be modified by drug exposure, and altered BDNF expression may influence conditioned behaviors.

In this study, we investigated the hypothesis that nicotine enhances the incentive salience of reward-predictive stimuli differentially by sex, and that nicotine’s effect is mediated by BDNF protein in key corticolimbic brain regions. Based on our previous studies, we predicted that nicotine exposure would increase the expression of CRs in both sexes, but with greater effects in females compared to males. In addition, we predicted that BDNF levels would be enhanced in nicotine-exposed compared to control-exposed animals.

## Methods

### Animals

A total of 24 male and 24 female Sprague-Dawley rats (225–250 g males, 174–190 g females on arrival) were purchased from Envigo (Indianapolis, IN, USA). Upon arrival, animals were housed in same-sex pairs in a vivarium on a 12:12 h light:dark cycle, and behavioral sessions were run during the light cycle. Throughout the experiment, rats were provided with food and water ad libitum. This experiment was conducted in accordance with the NIH Guide for the Care and Use of Laboratory Animals and approved by the Institutional Animal Care and Use Committee of the University of North Carolina at Chapel Hill.

### Behavioral training

Behavioral training occurred during Pavlovian conditioning sessions described previously [22]. Prior to training, pair-housed animals were given access to the 20% sucrose (*w*/*v*) solution that would be used as the US. Two sipper bottles containing the sucrose solution were placed in the home cage for 1 h, and rats were monitored by the experimenter to confirm that each rat consumed some of the solution. Next, each pair of rats were randomly assigned to a nicotine (NIC) or saline (SAL) drug exposure group and were habituated to the injection procedure with a single injection of the assigned drug on two consecutive days. NIC rats received 0.4 mg/kg nicotine hydrogen tartrate salt (Sigma-Aldrich, St. Louis, MO; subcutaneously, calculated using the free base form), dissolved in sterile saline with the pH adjusted to 7.0 ± 0.2. SAL animals received an equivalent volume of saline.

Prior to Pavlovian conditioning sessions, animals were introduced to the testing chambers during a magazine training session. For this session, rats were injected with the assigned solution 10 min before being placed into an operant chamber (MedAssociates, St Albans, VT). Each chamber was assembled with a stimulus light, retractable lever, and recessed reward receptacle on one wall of the chamber and a house light on the opposite wall. Animals remained in the testing chamber for 5 min before session initiation; thus, the nicotine or saline injection occurred 15 min prior to the start of the session. During this training session, the house light was illuminated throughout the session and animals received 15 deliveries of the US (0.1 ml of the 20% sucrose) into the reward receptacle on a variable interval 120-s schedule of reinforcement. Head entries into the receptacle were recorded by a photobeam detector but had no programmed consequences.

After magazine training, all animals underwent 29 Pavlovian conditioning sessions. These sessions were initiated as described above, with injections occurring 10 min before placement in the testing chamber for an additional 5 min before the session start. Each session was comprised of 15 CS-US pairings. The CS was a compound stimulus, consisting of extension of a retractable lever and illumination of a stimulus light directly above the lever; it was presented for 30 s on a variable interval 120-s schedule. Animals were able to interact with the lever during cue presentation, and both lever presses and receptacle entries were recorded, but had no programmed consequences. After the CS presentation, the stimulus light extinguished, the lever retracted, and the US (0.1 ml of 20% sucrose) was delivered into the receptacle.

### Tissue processing and Western blot procedure

Approximately 24 h after the final Pavlovian conditioning session, animals were euthanized and brains were collected for Western blotting. Animals were rapidly decapitated without anesthesia by trained personnel, and brains were removed and flash frozen in isopentane cooled with dry ice. Brains were stored at − 80 °C before processing. Tissue punches of each region of interest [orbitofrontal cortex (OFC), nucleus accumbens (NAc), and basolateral amygdala (BLA)] were taken from 300 μm coronal sections on a cryostat using a 1-mm tissue punch (Miltex, York, PA). Samples were diluted in homogenization buffer [1% sodium dodecyl sulfate (SDS), 10 mM Tris (pH 7.4), protease inhibitor cocktail tablets (Roche, Indianapolis, IN)], homogenized using a sonicator probe, and then centrifuged at 4 °C for 15 min at 12,000 *× ɡ*. The supernatant was collected and protein concentration was determined using the Pierce BCA assay (Thermo Fisher, Waltham, MA).

Twenty micrograms of protein were diluted in Laemelli sample buffer (Bio-Rad, Hercules, CA) and boiled at 95 °C for 5 min before being loaded on a precast 4–15% Tris-glycine gel (Bio-Rad) for SDS-polyacrylamide gel electrophoresis in Tris/glycine/SDS running buffer. Samples from all treatment groups (Male/Female, NIC/SAL) were included on each gel. Proteins were transferred to a polyvinylidene fluoride membrane using the Trans-Blot Turbo Blotting System (Bio-Rad) with transfer settings for mixed molecular weight proteins. Membranes were blocked in blocking solution containing 5% nonfat milk in Tris-buffered saline with 0.1% Tween 20 (TBST) for 1 h at room temperature, and then incubated at 4 °C overnight with primary antibodies against BDNF [ab108319 rabbit anti-BDNF, 1:1000 (Abcam, Cambridge, MA)] or GAPDH [MA5–15738 mouse anti-GAPDH, 1:1000 (Thermo Fisher)] in 1% blocking solution. Membranes were washed in TBST and then incubated with secondary antibodies (HRP-conjugated donkey anti-rabbit or goat anti-mouse, 1:5000) in 1% blocking solution for 2 h at room temperature. Enhanced chemiluminescence substrate (Bio-Rad) was added and blots were imaged using the ChemiDoc Imaging System (Bio-Rad). Bands for the mature form of BDNF were visible at 15 kDa and for GAPDH at 37 kDa. Quantification of band intensities was completed using Bio-Rad Image Lab software.

### Statistical analysis

Analysis of behavioral responses was completed using SigmaPlot v11.0 software (Systat Software Inc., San Jose CA). The last 10 days of training were averaged and compared between male and female rats exposed to nicotine or saline using a two-way ANOVA followed by Tukey’s HSD for post-hoc comparisons. Behavioral dependent measures were latency to press the lever (i) or enter the reward receptacle (ii) during the 30-s cue presentation, lever presses per trial (iii), a receptacle elevation score (iv), and the probability of entering the receptacle (v) or pressing the lever (vi) during a trial. Receptacle elevation scores were calculated by subtracting the number of receptacle entries that occurred during the 30-s period before a trial began from the number of receptacle entries that occurred during the 30-s trial [18, 22]. The probability of a lever press or receptacle entry was calculated as the number of trials in which the behavior occurred, divided by the total number of trials in a session. For all analyses, α was set at 0.05. Effect sizes were considered small, medium, or large if they corresponded to partial η^2^ of at least 0.0099, 0.0588, and 0.1379, respectively, based on values of ƒ as described by Cohen [49]. To compare day-to-day variability between sexes, a coefficient of variation was calculated for each behavioral measure for each rat across the last 10 days of training [25, 50] and analyzed by two-way (sex*drug) ANOVA.

We calculated a Pavlovian conditioning score to categorize animals as goal-trackers, sign-trackers, or intermediate animals [25]. This score considers the above-mentioned measures of conditioned responding as well as the number of trials in which the animal first interacts with the CS or US (designated as CS and US trials, respectively). The formula for this tracking score is as follows:

$$ \frac{\left(\frac{\mathrm{lev}.\mathrm{press}.-\mathrm{elev}.\mathrm{score}}{\mathrm{lev}.\mathrm{press}.+\mathrm{abs}.\mathrm{value}\ \mathrm{of}\ \mathrm{elev}.\mathrm{score}}\right)+\left(\frac{\mathrm{recept}.\mathrm{latency}-\mathrm{lev}.\mathrm{latency}}{30}\right)+\left(\frac{\mathrm{CS}\ \mathrm{trials}-\mathrm{US}\ \mathrm{trials}}{\mathrm{CS}\ \mathrm{trials}+\mathrm{US}\ \mathrm{trials}}\right)}{3} $$

Scores from 0.3 to 1.0 were classified as sign trackers, while scores from − 0.3 to − 1.0 were classified as goal trackers, and scores from − 0.3 to 0.3 were classified as intermediate.

BDNF protein as detected by Western blots was normalized to the GAPDH loading control. To allow for comparisons between both sex and drug exposure groups, all values were normalized to female SAL controls within each gel and then compared by two-way (sex*drug) ANOVA.

## Results

### Pavlovian conditioned approach

**Fig. 1:**
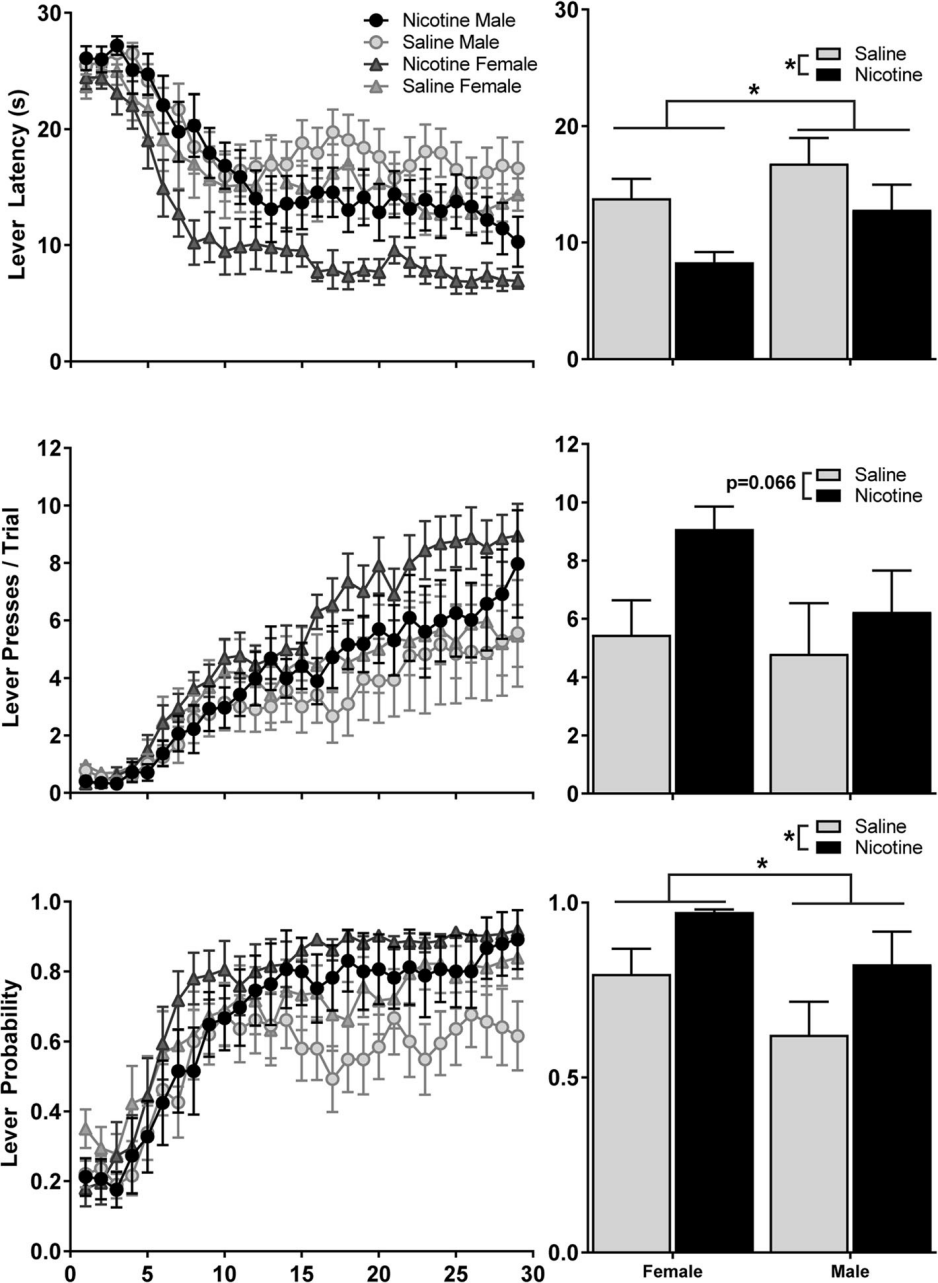
Nicotine and female sex enhance sign-tracking in rats. Expression of sign-tracking behaviors over 29 days of training (left) and averaged across the last 10 days of training (right) in male and female rats that received nicotine injections prior to each session, compared to saline-injection control groups. Data are expressed as mean ± SEM, and reflect separate measures of conditioned approach to the conditioned stimulus: Top latency to press the lever; Middle lever presses per trial; Bottom probability of pressing the lever during a trial. * Main effect of nicotine exposure or sex, *p* < 0.05

**Fig. 2:**
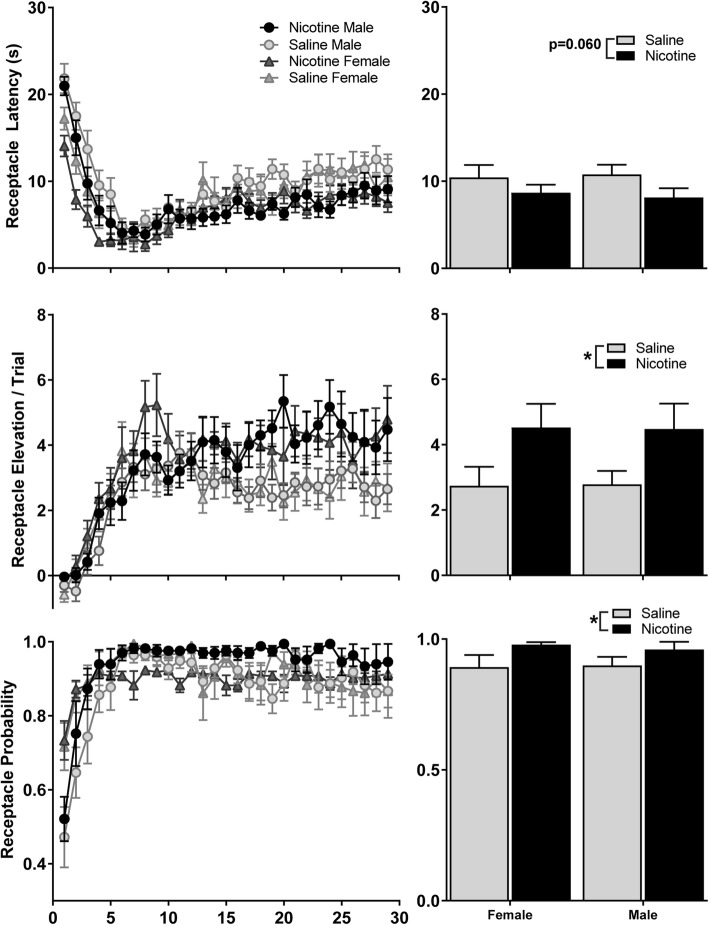
Nicotine enhances goal-tracking in male and female rats. Expression of goal-tracking behaviors over 29 days of training (left) and averaged across the last 10 days of training (right) in male and female rats exposed to nicotine or to saline. Data are expressed as mean ± SEM, and reflect separate measures of conditioned approach to the reward receptacle: Top latency to enter the receptacle; Middle receptacle elevation score per trial; Bottom probability of entering the receptacle during a trial. * Main effect of nicotine exposure, *p* < 0.05

Both male and female animals successfully formed an association between CS presentation and subsequent US delivery, regardless of drug exposure, as shown by an increase in conditioned responding during the 29 days of training in all groups (Figs. 1 and 2). Nicotine exposure enhanced both sign- and goal-tracking CRs, and females showed elevated sign-tracking on some measures. For sign-tracking behaviors (Fig. 1), there was a marginal effect of nicotine exposure on lever presses (*F*_1,47_ = 3.5, η^2^ = 0.07, *p* = 0.066), and significant main effects of exposure on latency to press the lever (*F*_1,47_ = 6.8, η^2^ = 0.12, *p* = 0.01) and probability of pressing the lever (*F*_1,47_ = 6.2, η^2^ = 0.11, *p* = 0.02). No difference between male and female animals emerged for number of lever presses, (*F*_1,47_ = 1.7, *p* = 0.20) but females pressed the lever faster than males (main effect of sex: *F*_1,47_ = 4.3, η^2^ = 0.08, *p* = 0.04) and were more likely to press the lever than males (main effect of sex: *F*_1,47_ = 4.5, η^2^ = 0.08, *p* = 0.04). Nicotine exposure also enhanced expression of goal-tracking behaviors in both male and female animals (Fig. 2). Nicotine exposure significantly increased receptacle elevation score (*F*_1,47_ = 7.7, η^2^ = 0.15, *p* = 0.008) and probability of entering the receptacle (*F*_1,47_ = 5.1, η^2^ = 0.10, *p* = 0.03), and marginally reduced receptacle latency (*F*_1,47_ = 3.7, η^2^ = 0.08, *p* = 0.06). We detected no main effect of sex on receptacle elevation, receptacle latency, or receptacle probability (all *F*_1,47_ < 0.1, all *p* > 0.80). There were no significant interactions between sex and drug exposure on any CR measures.

In addition to investigating sex and drug-exposure differences on each CR measure, we computed a Pavlovian conditioned approach score to categorize animals as sign-trackers, intermediates, or goal-trackers (Table 1). While the saline-exposed female group exhibited equal numbers of sign-tracking and goal-tracking rats, the nicotine-exposed female group contained only intermediate and sign-tracking phenotypes. In contrast, nicotine-exposed males were more likely to be classified as intermediate, while saline-exposed males were likely to be sign trackers or goal trackers.

**Table 1:** Distribution of sign- and goal-tracking animals by sex and drug exposure (SAL = saline, NIC = nicotine). A tracking score (see text) was calculated for each rat, based on conditioned approach behavior on the last 4 days of training. The score was used to classify rats within sex and drug exposure group as goal-trackers, intermediate, or sign-trackers

	Goal-tracker	Intermediate	Sign-tracker
SAL Female, *n* = 12	25%	50%	25%
NIC Female, *n* = 12	0%	58%	42%
SAL Male, *n* = 13	46%	31%	23%
NIC Male, *n* = 11	9%	82%	9%

We did not measure estrous stage in this study, but if estrous cycle influenced conditioned approach, it follows that females would show more day-to-day variability as compared to males. To assess individual variability by sex, we calculated the coefficient of variation (CV) across the last 10 days of training for male and female rats in both the NIC and SAL groups (Table 2). A main effect of sex emerged for lever latency (*F*_1,47_ = 4.2, η^2^ = 0.1, *p* = 0.05) where females showed a higher CV than males. A trend toward a main effect of nicotine exposure also emerged for lever latency (*F*_1,47_ = 3.4, *p* = 0.07), with nicotine-exposed animals demonstrating a slightly higher CV than SAL rats. There was also a trend toward a main effect of sex for lever probability (*F*_1,47_ = 3.4, p = 0.07), where males showed a slightly higher CV than females. In addition, a main effect of drug exposure emerged for receptacle probability (*F*_1,47_ = 6.5, η^2^ = 0.15, *p* = 0.01), in which SAL animals had a higher CV than NIC rats. Other main effects and interactions did not reach significance (all *F*_1,47_ < 1.6, all *p* > 0.15). Thus, both sex and nicotine appeared to influence day-to-day variability in isolated aspects of CRs, but not in a systematic manner.

**Table 2:** Individual variability in behavior by sex and drug exposure (SAL = saline, NIC = nicotine) across the last 10 days of training. The coefficient of variation (see text) was calculated for each animal and averaged across groups for each sign-or goal-tracking behavior (presented as mean ± SEM)

	Lever latency^a^	Lever press	Lever probability	Receptacle latency	Receptacle entries	Receptacle probability^b^
SAL Female	26.6 ± 2.9	40.1 ± 10.3	20.0 ± 5.8	34.6 ± 4.7	27.8 ± 3.4	10.3 ± 3.3
NIC Female	28.1 ± 2.2	21.7 ± 2.1	4.6 ± 0.9	30.4 ± 2.2	22.4 ± 2.4	2.4 ± 0.9
SAL Male	17.6 ± 2.2	44.8 ± 8.4	30.2 ± 7.9	29.4 ± 2.9	29.4 ± 2.9	11.1 ± 3.4
NIC Male	26.1 ± 3.6	45.6 ± 15.1	25.7 ± 14.7	29.9 ± 2.6	24.0 ± 4.5	5.0 ± 2.7

^a^Main effect of sex (*p* < 0.05), with females more variable than males (collapsed across exposure)

^b^Main effect of exposure (*p* < 0.05), with SAL groups more variable than NIC groups (collapsed across sex)

### Western immunoblot for BDNF

**Fig. 3:**
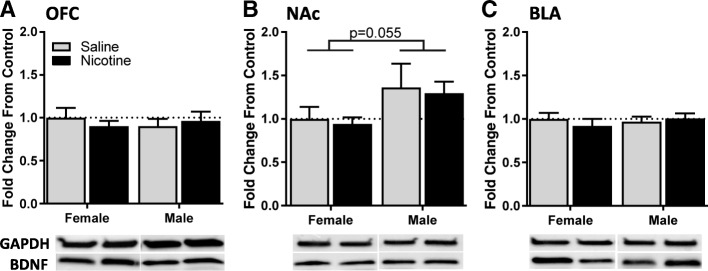
BDNF protein levels in the OFC, NAc, or BLA after nicotine exposure. BDNF protein was normalized to GAPDH loading control and expressed as a proportion of female saline controls. Protein was measured in the **a** OFC, **b** NAc, and **c** BLA. Representative bands of GAPDH and BDNF protein are presented for each region, aligned with their respective groups in the above bar graphs

Tissue from the OFC, NAc, and BLA was analyzed by Western blot to measure levels of BDNF protein between sexes and after prolonged nicotine exposure (Fig. 3). To allow direct comparisons between sex and drug by two-way ANOVA, BDNF protein samples were normalized to female saline controls within each gel. While no main effects of nicotine exposure on BDNF reached significance for males or females in any brain region, BDNF levels in the nucleus accumbens were marginally higher in males than in females (*F*_(1, 47)_ = 3.9, *p* = 0.055). No other main effects (drug, sex) or sex*drug interactions emerged (all *F*_1,47_ < 0.79, all *p* > 0.37).

## Discussion

This study tested the hypotheses that nicotine exposure would increase expression of CRs, especially in females, and that this difference would be reflected in altered BDNF protein levels. Supporting our hypothesis, we report that females exhibited more sign-tracking CRs than males and that nicotine enhanced the expression of both sign-tracking and goal-tracking in females. We also replicated previous reports that nicotine enhances conditioned approach in males [18, 22, 23]. Contrary to our predictions, we did not find nicotine-associated changes in BDNF protein levels in the OFC, NAc, or BLA, although we did discover that BDNF expression was slightly higher in males than females. While this finding may be related to the observed sex differences in behavior, the larger data set suggests that BDNF expression in these brain regions is not strongly associated with other aspects of conditioned approach or nicotine exposure in general.

The impact of sex on CRs described here is in line with prior findings from our lab [25], though others find limited effects of sex on CRs [26]. Our lab recently observed that female rats exhibited more sign-tracking CRs and were more likely to be classified as sign trackers in a study that investigated conditioned approach after adolescent intermittent ethanol exposure [25]. While the present study is consistent with our prior one, the magnitude of sex differences was larger in Madayag et al. [25]. In that study, rats were bred in-house while the present study used vendor supplied rats, suggesting that litter size or early life environment may influence the difference in magnitude of observed sex effects.

The present findings support the importance of inclusion of both male and female animals when measuring behavior [50, 51]. In some cases, drug exposure can influence females differently than males, despite a lack of behavioral disparity in drug-naïve conditions. For example, nicotine causes sex-dependent effects in both preclinical and clinical populations [52, 53]. Female rodents are sensitive to nicotine-associated stimuli, while males are more sensitive to the interoceptive effects of nicotine [32], and studies in male and female human smokers have also supported the possibility of similar sex-differences in response to nicotine [54]. This enhanced sensitivity in females to conditioned cues may underlie the elevated sign-tracking behavior observed among female rats in the present experiment. Of note for this study, sex differences in the locomotor-activating effects of nicotine may not be present in rodents [55, 56], suggesting that the decreased latency to contact the lever observed in females was primarily due to the incentive motivational properties attributed to the salient CS, rather than to sex-specific hyperactivity. We also note that both females and males primarily chose to approach the receptacle before the lever, and no sex differences emerged for this measure, further indicating that females were not simply hyperactive. Given that females demonstrated increased sign-tracking behaviors across multiple measures, it appears that females are more likely to perform this behavior than males regardless of nicotine exposure.

Although we did not directly measure estrous cycle phase, which would have enabled us to draw conclusions about putative cycle effects on behavior, published data indicate that estrous cycle does not affect sign- and goal-tracking behavior in females [26]. To evaluate day-to-day variability in behavior that could be attributed to the 4-day rodent estrous cycle, we analyzed the coefficient of variation over the last 10 days of training (encompassing at least 2 cycles). We found that for the majority of behavioral measures, female rats were not more variable than males, replicating our previous study [25]. The one measure for which females showed higher variability than males was lever latency where females were faster than males to press the lever, or sign track, over the same time period. Conversely, we found a trend in the opposite direction for lever probability with males exhibiting more variability than females on this measure, even though behaviorally females showed an enhanced probability of pressing the lever over this same time period. No other sex differences in day-to-day variability emerged on behavioral measures. Thus, the data do not support the interpretation that hormonal variations due to the estrous cycle are the cause of the behavioral differences between males and females in this study.

We examined BDNF levels in the NAc, OFC, and BLA—corticolimbic brain regions known to be involved in Pavlovian conditioning (e.g., [57, 58]). Contrary to our hypothesis, we found no effect of nicotine exposure on BDNF protein in the OFC, BLA, or NAc 24 h after the final injection. We detected a slight sex difference in that male animals had higher BDNF in the NAc than females, but no differences emerged in the other regions tested. Sex differences in BDNF have been reported in brain regions such as the hippocampus and the prefrontal cortex, often after stress [59–61]. Developmental manipulations of steroid hormones can have lasting impact on BDNF levels [62, 63], and suggest a mechanism for the emergence of sex differences in BDNF observed in adulthood. The slight sex difference in BDNF protein detected in this study is of interest as the NAc integrates inputs from cortical and limbic structures to promote motivated behaviors of relevance to addiction. The NAc is a significant location where sex differences in BDNF may influence sex differences in other addiction-associated behaviors [64]. The specific link between sex and BDNF in the NAc, driven by organizational or circulating hormonal effects, can be further analyzed in future studies that directly measure baseline sex differences in BDNF and the impact of drug exposure.

Although others have found differences in BDNF expression based on sign- or goal-tracker classification, with sign-trackers having less BDNF expression in the prefrontal cortex compared to goal-trackers [37], we did not see such an effect in a prefrontal subregion (OFC), nor did we observe nicotine effects on BDNF in the NAc or BLA. We expected altered BDNF protein expression among nicotine-exposed animals, given data showing increases or decreases in corticolimbic BDNF after acute or chronic nicotine exposure, respectively [46–48]. Prior studies that detected nicotine-induced changes in BDNF focused on regions such as the hippocampus [45, 65], ventral tegmental area [46], and dorsal striatum [47] that were not tested here. Thus, it may be that alterations in BDNF in brain regions not assessed in the present study contribute to the behavioral effects of nicotine on Pavlovian approach behavior.

These previous studies also used nicotine exposure regimens that yielded higher doses of nicotine over either the acute period or after a chronic period, which stimulated persistent changes in behavior and synaptic plasticity. In the present study, we assessed BDNF protein 24 h after the final injection to avoid measuring acute effects of nicotine or behavioral training on BDNF protein, and instead to measure longer-lasting effects of multi-day drug exposure. In rats trained with the same nicotine dose and schedule, we previously reported that substituting saline for nicotine before a Pavlovian conditioning session resulted in a reduction in behavior that returned the nicotine-enhanced CRs to the level of control animals [22]. Furthermore, at blood plasma levels relevant to human cigarette inhalation, self-administered nicotine increases sign-tracking behaviors, but this effect may be transient and dissipate when nicotine is no longer on board, despite an extensive history of drug exposure [24]. Thus, it is possible that despite 30 days of nicotine exposure, the nicotine dose in this study was not sufficient to elicit changes in BDNF expression measurable after 24 h.

Similarly, it is possible that alterations in BDNF occurred at an earlier time-point during training. At the time of tissue collection in the current study, animals were well-trained on the Pavlovian approach paradigm and expressed stable conditioned approach behavior. BDNF is involved in synaptic plasticity as well as learning and memory [66–68], and differences in expression may have occurred at the time of initial acquisition of the conditioned approach behavior. A future test of BDNF protein expression related to behavior and nicotine exposure could incorporate new learning, such as extinction training or a reversal task, to challenge the ability of sign- and goal-tracking rats to update their behavior after a change in stimulus-reward association.

## Conclusions

This study presented evidence that nicotine enhances conditioned approach in females similarly to its known effect in males. In addition, we add to the growing literature of sex differences in reward conditioning, demonstrating that females show an increased likelihood of expressing sign-tracking compared to males. BDNF protein in mesocorticolimbic brain regions, proposed to be involved in this behavior, was not influenced by conditioned responding or nicotine exposure.

## Authors’ information

SJS is currently at the Department of Psychiatry, University of Pittsburgh, Pittsburgh, PA, USA. ACM is currently at NeuroCycle Therapeutics, Milwaukee, WI, USA.
